# The core of the matter: How do scientists judge trustworthiness of physical samples?

**DOI:** 10.3389/frma.2022.1034595

**Published:** 2022-10-28

**Authors:** Peter Thomas Darch

**Affiliations:** School of Information Sciences, University of Illinois at Urbana-Champaign, Champaign, IL, United States

**Keywords:** open data, open science, subseafloor biosphere, physical samples, data curation, data management, knowledge infrastructures, metadata

## Abstract

In recent years, research funding agencies, universities, and governments have become increasingly concerned with promoting the reuse of research datasets. Enabling researchers to evaluate the trustworthiness and fitness-for-use of research datasets produced by others is critical for facilitating the reuse of these datasets. Understanding how researchers make these evaluations is crucial for developing digital infrastructure and tools, such as data repositories and metadata schema, in a way that better supports researchers in making these evaluations. Physical samples such as rocks are critical for generating datasets in many scientific domains. Often, samples are collected on field expeditions conducted by large infrastructural projects. These projects comprise many human and non-human components that affect the quality and integrity of samples. However, little is known about whether and how prospective dataset users evaluate the samples' trustworthiness and sample collection processes underlying these datasets. Researchers‘strategies for evaluating sample trustworthiness are explored through a longitudinal qualitative case study (ethnographic observation, interviews (*n* = 66), and document analysis) of subseafloor biosphere research, an earth sciences domain. Domain researchers use rock samples collected on research cruises conducted by the International Ocean Discovery Program (IODP). Subseafloor biosphere researchers are primarily concerned about samples being compromised by microbiological contamination. Researchers vary regarding the components of IODP infrastructure they consider when evaluating sample trustworthiness. These components include methods to process samples, people handling samples, IODP policies and procedures, and IODP organizational politics. Researchers‘strategies vary according to their disciplinary background, with microbiologists employing more fine-grained judgments about methods; whether researchers have participated in IODP expeditions, with those who have employing more fine-grained judgments about people involved; and whether researchers have ever been involved in organizing cruises or serving on IODP committees, with those who have employing more fine-grained judgments about many aspects of cruises. Researchers who make less complex decisions may be prone to erroneously trusting contaminated samples; researchers who make more complex decisions may be prone to erroneously discarding uncontaminated samples. The paper concludes by considering implications for the design of digital infrastructures to support researchers in evaluating sample trustworthiness.

## Introduction

A critical concern when building digital infrastructures for research dataset curation is how to support researchers in evaluating the integrity and trustworthiness of the datasets they want to use (Faniel and Jacobsen, 2010; Rolland and Lee, 2013). In many research domains, such as those in the earth sciences, datasets are often generated by analysis of physical samples (McNutt et al., 2016). However, these samples are frequently invisible to users of digital data curation infrastructure. This paper shines a light on these physical objects, tracing concerns about datasets back to the integrity and trustworthiness of the underlying samples and considering the implications for designing digital infrastructures to make these dimensions of samples more visible to researchers as they evaluate samples for use.

In the earth sciences, critical physical samples include cores (long sections of rock extracted from the ground or seafloor). However, samples are often scarce, expensive and time-intensive to collect and store. If not curated appropriately, samples may get lost or suffer irreversible damage. Key stakeholders, including researchers and federal funding agencies, seek improved curation of physical samples (Council, 2002; Ramotnik, 2015; Karadkar et al., 2017). Initiatives for sample curation in the earth sciences have focused mainly on the findability and accessibility of samples (Devaraju et al., 2017; Lehnert, 2017; Lehnert et al., 2019). However, prospective users of samples also need to trust in the integrity of samples and be confident that resultant datasets provide an accurate representation of conditions in the environment where the sample originated.

Researchers face challenges when judging a sample's trustworthiness. In many cases, researchers procure samples from large-scale infrastructural projects comprising many components: human (e.g., professional staff), physical (e.g., tools, laboratories), and intangible components (e.g., methods). Each component may enhance or compromise a sample's integrity. Exhaustively gathering information about all components is impossible for a prospective sample user: instead, the prospective user must judge sample integrity with incomplete information.

Understanding how researchers make such judgments enhances possibilities to facilitate sample use and reuse. Upon what components of the system that produced the sample do researchers focus? What information do researchers use to make judgments about these components? How do researchers differ in how they make judgments? Answers to these questions can inform measures, such as including information about provenance in online sample databases, to support researchers' existing ways of making judgments.

This paper addresses these issues through the case of researchers who study microbial communities in the seafloor and interactions between these communities and the physical environment they inhabit. Deep subseafloor biosphere research is part of the subfield of earth sciences called biogeosciences. This paper focuses on how deep subseafloor biosphere researchers judge the trustworthiness of samples drawn from cores procured from scientific ocean drilling cruises operated by the International Ocean Discovery Program (IODP).

Researchers are especially concerned about whether samples are at risk of biological contamination. Researchers want to be able to trust and use samples and are pragmatic, often improvising trust strategies. Strategies differ between researchers due to multiple factors. One is the level of involvement in IODP cruises. Those who have never sailed on IODP cruises struggle to make even rudimentary judgments about sample trustworthiness. In contrast, those who have sailed employ more detailed judgments about the people and methods involved. Some researchers have been involved in either organizing IODP cruises or serving on IODP policymaking committees. These researchers consider the effect on sample integrity of politics between different domains served by IODP. Researchers' disciplinary backgrounds and identities also play a significant role in shaping trust strategies. Researchers with microbiological backgrounds tend to make more detailed judgments of methods to mitigate and assess contamination than those with physical science backgrounds. Finally, the strength of researchers' professional networks also shapes researchers' trust strategies by enabling access to different types of provenance information about particular samples.

## Background

Collection, handling, management, and curation of samples require infrastructure. This section discusses infrastructures as they relate to the production of scientific knowledge, highlighting their technical, physical, social, and political dimensions. Attention then turns to the challenges of curation and circulation of physical samples, particularly in the earth sciences. While several studies and initiatives address the findability and accessibility of samples, far less attention has been paid to the issue of how prospective users of samples make judgments about sample trustworthiness. This section concludes by considering studies of how researchers make trust judgments about knowledge products other than samples, how these judgments intersect with questions of infrastructure, and how insights from these studies might apply to physical samples.

### Knowledge infrastructures

Star and Ruhleder (1996) theorized infrastructures as complex ecologies comprising multiple human and non-human elements. They characterize infrastructure as transparent and only becoming visible upon breakdown, which means that infrastructure and its inner workings are often invisible to its users.

Knowledge infrastructures are infrastructures that support the production and spread of scholarly knowledge (Edwards, 2010; Edwards et al., 2013). Cyberinfrastructure, including databases, is a key component of knowledge infrastructures, facilitating collaboration between geographically- and institutionally-distributed researchers and enabling the collection, management, and circulation of digital research objects such as datasets (Bietz et al., 2010; Ribes and Lee, 2010). Cyberinfrastructure involves human infrastructure (Jirotka et al., 2013), who possess technical and scientific skills and expertise. Sometimes their work is invisible to outsiders (Plantin, 2018), notably those who build and maintain technical systems (e.g., databases or research drilling cruises), technicians, and curators of research objects.

Knowledge infrastructures must often accommodate, adapt to, and be contested over the changing requirements of multiple scientific domains (Darch and Sands, 2017), and not all interests of all relevant stakeholders can be pursued equally (Ribes and Lee, 2010; Parmiggiani, 2017). However, changing infrastructure to accommodate changing requirements is often difficult, limited by the installed base of the preexisting infrastructure and the scarcity of available resources (Ribes and Lee, 2010; Parmiggiani, 2017).

The difficulties of adapting infrastructure may become especially fraught when the preexisting infrastructure is expected to serve a new scientific domain (Benson, 2012; Jackson and Buyuktur, 2014), as existing domains already served by the infrastructure may resist the requirements of the new domain, given the scarcity of infrastructural resources (Darch and Borgman, 2016).

### Challenges and initiatives for curating physical samples

Several studies and initiatives have sought to improve the curation of physical samples in the geosciences (Lehnert et al., 2014; Klump et al., 2018). These initiatives have paid little attention to supporting prospective users of samples to make judgments about sample trustworthiness, instead focusing on improving the discoverability and accessibility of physical samples and linking samples to associated datasets and publications. One example is a repository outlined in Devaraju et al. (2017). This repository builds upon other initiatives, such as International Geo Sample Numbers (ISGN), persistent and globally-unique identifiers assigned to samples (Lehnert et al., 2019). The repository also employs a metadata model developed by the Commonwealth Scientific and Industrial Research Organization to describe samples (Golodoniuc et al., 2016). This model includes elements such as the name of the sample's collector, and basic information about the sample's origin location, how it was collected, and its subsequent curation.

Improving the discoverability and accessibility of samples is necessary to improve the curation of samples and facilitate their use. Promoting the integrity and trustworthiness of samples is also an essential part of curation (Ramdeen, 2015). However, existing metadata schemas typically only allow for the provision of limited information about the conditions under which samples were collected and handled.

### Trust in knowledge products

While issues of trust regarding samples remain largely unaddressed, scholars have addressed trust in other knowledge products, most notably datasets (Rolland and Lee, 2013). Trust in knowledge products is critical to scientific practice (Hardwig, 1985, 1991). Wilholt (2013) breaks this epistemic trust into two dimensions: trust in the methods used to produce the knowledge product and trust in the individual(s) producing the knowledge product. The second of these dimensions breaks down further into the honesty and benign intent of the individual producing the knowledge product and that individual‘s competence in using appropriate methods and tools (Shapin, 1994).

**Figure 1:**
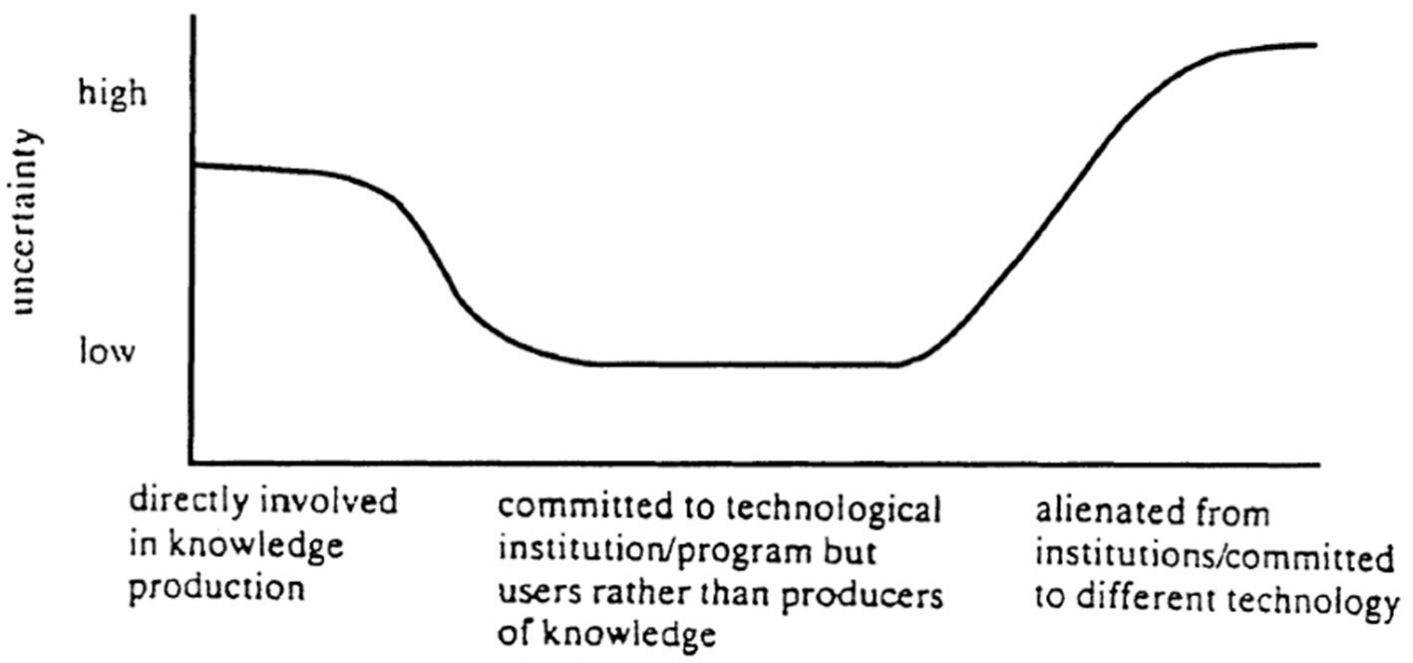
The certainty trough, reproduced from MacKenzie (1993).

However, knowledge products are often generated by large and complex infrastructures that comprise many human and non-human components. It is infeasible for anyone to make a trust judgment about a knowledge product by evaluating every one of these components (Giddens, 2013) for three reasons. One is that an individual would be overwhelmed by documenting and evaluating every component. A second is that an individual may be external to the system, thus unable to see the inner workings of the system. The third reason is that the system is likely to have a vested interest in obscuring the messiness, contingency, and contested nature of the knowledge claims it generates; instead, the system wants to promote trust in these claims by presenting them as objective. Latour (1987) labeled the process of obscuring the inner workings of a system that produces a knowledge claim as black-boxing. Successful black-boxing turns a knowledge claim into a scientific fact. Other scientific knowledge products may also be black-boxed, such as a dataset (when regarded as an objective representation of reality) and a method or set of processes for producing scientific knowledge products (when regarded as reliable and accurate). MacKenzie (1993) introduced the certainty trough to chart how an individual's trust in a technological or scientific system producing knowledge claims varied according to that individual's position in relation to that system (see Figure 1). An individual close to the center of the system, familiar with its inner workings' messiness, contingency, and politics, exhibits great uncertainty in the system, while an individual external to the system but supportive of the system exhibits greater certainty in the system.

Researchers need to trust in the accuracy, reliability, and validity of the datasets they wish to use (Kowalczyk and Shankar, 2011). The judgment of trustworthiness is a question of perception rather than determined by essential properties of the dataset (Prieto, 2009). Studies of how researchers assess dataset trustworthiness show that researchers often follow one of two strategies that broadly correspond to the two dimensions of epistemic trust discussed above. A researcher may also combine strategies (Yoon, 2017).

One strategy relates to trusting in the individual(s) who produced the dataset. Trust can be based on a producer's reputation (Jirotka et al., 2005) or whether the producer is a member of the appropriate community of practice: did they use suitable methods and modes of reasoning (Van House, 2002a,b)? Other studies reveal practices of evaluating information, or contextual metadata, about the contexts in which the dataset was produced and handled (Faniel and Jacobsen, 2010; Faniel and Yakel, 2011). Datasets pass through multiple stages before they arrive in the hands of their prospective user, from planning through collection, analysis, dissemination, and storage. Actions taken at each stage can impact a dataset's integrity (Borgman, 2019; Wing, 2019). Prospective dataset users need to trust the methods and instruments used to handle datasets (Borgman et al., 2012). And these prospective users may consult contextual metadata for information about these methods and instruments. Using contextual metadata can be difficult. Adequate metadata are often not recorded when actions are taken on datasets (Schuurman and Balka, 2009). Challenges of trusting datasets multiply when datasets are transferred across disciplinary boundaries as researchers may need to interpret and validate data collected in methodological and epistemological contexts with which they are unfamiliar (Young and Lutters, 2017).

Other studies have focused on trust in repositories and databases as complex systems. Yoon (2013) found that users' perceptions of digital repositories' trustworthiness were influenced by actions, such as quality checking and provision documentation, taken by the repository, as well as being shaped both by the user's past experiences and by a repository's reputation in the user communities. In a study comparing archaeologists' and social scientists' use of data repositories, Yakel et al. (2013) found variations between disciplines regarding what factors shaped users' trust in the repositories.

### Research questions

Improving physical sample use promises many benefits for earth science research. As with other knowledge products, a prospective sample user must be able to trust that sample. To support and improve researchers' trust strategies in physical samples, this paper addresses the following research questions:

1) Against what criteria do prospective users of physical samples judge the trustworthiness of these samples?
2) What information do these prospective users use to support their trust judgments?
3) What underlying factors shape researchers' trust judgments?

### Deep subseafloor biosphere research

These research questions are addressed by studying an earth sciences research domain that relies on physical samples, deep subseafloor biosphere research. Domain researchers integrate physical science and bioinformatics data to answer questions about relationships between microbial communities in the seafloor and the physical environment they inherit.

Studies of the deep subseafloor biosphere began in the late 1990s. Two infrastructures have been instrumental to this domain's emergence. One is the Center for Dark Energy Biosphere Investigations (C-DEBI), a ten-year NSF Science and Technology Center launched in 2010, providing funding to over 150 researchers across the US and Europe (C-DEBI, 2019). The second infrastructure is the International Ocean Discovery Program (IODP), which operates five scientific ocean drilling cruises per year to procure cores of the seafloor, from which samples are taken for analysis (IODP, 2019). IODP serves multiple domains besides deep subseafloor biosphere research, such as studies of plate tectonics. While IODP and its precursors began in the 1960s, subseafloor biosphere research has only been included in IODP activities since the early 2000s. As with other infrastructures built to enable seafloor research (Parmiggiani et al., 2015), IODP must resolve the interests of many different groups of stakeholders as a single cruise cannot serve the needs of all of IODP's constituent scientific domains. A critical driver of C-DEBI's foundation was helping deep subseafloor biosphere researchers intervene more successfully in IODP politics around allocating resources between competing research domains; conversely, the outcome of these politics shape C-DEBI project activities (Darch and Borgman, 2016).

Researchers' work requires access to high-quality physical samples from the seafloor procured on research cruises. However, the cost and logistics of these cruises mean they are rare. Researchers frequently rely on samples that they have not collected themselves. Deep subseafloor biosphere research affords rich opportunities for characterizing trust practices concerning physical samples. The author‘s engagement with this community and data collection began in August 2012 and continues until this day.

## Methods

Data collection comprised ethnographic observation, interviews, and document analysis (Hammersley and Atkinson, 2007). Scholars have successfully applied these methods to study scientific work (Latour, 1979; Traweek, 1988; Knorr-Cetina, 1999). They have recently adapted them to study knowledge infrastructures (Ribes and Lee, 2010; Karasti et al., 2018). Knowledge infrastructures have many features that pose challenges to the analyst, and how standard qualitative methods were adapted to address these challenges is discussed here. The work of building and using infrastructures and establishing and sustaining distributed collaborations is typically spread across many sites and time, often on the scale of years or decades (Karasti et al., 2010).

Working under time and resource constraints, the analyst has to make tough choices about what to include within the boundaries of their field site (Parmiggiani, 2017). One choice was to study two knowledge infrastructures (C-DEBI and IODP) and their relationships, given their critical interdependence.

Ethnographic observation of subseafloor biosphere researchers and IODP personnel allowed the author to observe how researchers made trust judgments about physical samples. It enabled the author to observe the tacit dimensions of these judgments that might not be described during an interview. The author also observed how knowledge relevant to making trust judgments about samples circulates among researchers.

An analyst studying a distributed collaborative project or infrastructure must address a key tension in allocating their attention between multiple sites. Deep engagement with a single site is critical for understanding how work is conducted. However, the analyst must also understand the roles and interactions between the many sites where work occurs (Star, 1999). The author resolved this tension by combining long-term observation of a single laboratory with several shorter-term periods observing other work sites. The author was embedded for eight months in a laboratory in the USA, headed by a leading figure in subseafloor biosphere research, visiting the laboratory for two or three days per week and observing meetings and bench work. The author also spent two weeks in two other US laboratories, joined researchers on short-term field research expeditions, made three trips to the International Ocean Discovery Program (IODP) offices and the core repository (seven days of observation in total), and attended and presented at academic meetings attended by subseafloor biosphere researcher (24 days total). These meetings included five annual American Geophysical Union Fall Meetings, which enabled the author to see the full range of research facilitated by the IODP and meet IODP representatives.

The in-person observation was supplemented by online observation (Hine, 2008). As distributed infrastructures, much of the work of IODP and C-DEBI is conducted online. The author observed IODP online meetings and seminars where cruises' scientific activities were planned and other workshops and meetings involving C-DEBI personnel. A feature-length documentary based on a subseafloor biosphere-focused IODP expedition gave insights into onboard activities.

Another component of this research is semi-structured interviews (*n* = 66) conducted with subseafloor biosphere researchers and IODP personnel in the USA, East Asia, and Europe. Some interviews were conducted in person; others over Skype. Interviews ranged from 35 min to two and a half hours, with a median length of 75 min. Table 1 summarizes the interview population.

**Table 1:** The composition of the interview sample (^*^N.B. regarding disciplinary background, most subseafloor biosphere researchers describe themselves as being at the interface of the life and physical sciences. They were allocated to “Life sciences” or “Physical sciences” in this table based on their primary background prior to starting work on subseafloor biosphere research).

		**Total**	**Gender**		**Geographic location**			Disciplinary background*	
			**Male**	**Female**	**USA**	**Europe**	**East Asia**	**Life sciences**	**Physical sciences**
Subseafloor	Undergraduate	**5**	1	4	5	-	-	4	1
biosphere	Graduate student	**9**	4	5	9	-	-	5	4
research	Postdoctoral researcher	**7**	4	3	6	1	-	5	2
	Faculty	**23**	17	6	12	8	3	18	5
	Management/organizational	**4**	1	3	4	-	-		
IODP	Cruise support	**13**	9	4	13	-	-		
	Management/organizational	**5**	4	1	5	-	-		
	**Total**	**66**	**40**	**26**	**44**	**9**	**3**	**32**	**12**

The bold values are the column/row totals (i.e., there were 5 undergraduates total).

Interview questions covered topics including interviewees' disciplinary backgrounds and training, their work, and their collaborative relationships. Subseafloor biosphere researchers were also asked to discuss how they used physical samples in their work, how they procured and evaluated these samples and their experiences with IODP cruises and policymaking. IODP personnel were asked to describe their decisions in building, operating, and maintaining infrastructure to support collecting and curating physical samples.

Interviews were recorded and transcribed, with two exceptions at the request of interviewees. Quotations from interviews are used in this paper. These quotations are anonymized but are assigned a code to indicate the interviewee's career stage and a randomized number unique to each interviewee: e.g., (Doctoral Student, #5). Finally, the author also collected a corpus of documents for analysis. These documents include operating documents for C-DEBI and IODP, including proposals, work plans and reports, and articles produced by researchers.

Initial analysis of these data comprised close reading of ethnographic notes, interview transcripts, and documents. From these readings, themes were identified. The coding scheme was refined iteratively. This human subjects research was approved by the UCLA North General Institutional Review Board (#10-000909-CR-00002).

## Results

This section presents the wide array of trust judgments made by subseafloor biosphere researchers about physical samples. These researchers use many types of physical samples (e.g., loose rocks from the seafloor, water) from expeditions carried out under the auspices of various organizations. However, for clarity, the focus here is on one type of sample (taken from cores, lengths of rock from the seafloor procured by drilling), produced on cruises operated by one organization (IODP), for one purpose (characterization of microbial communities in the sample). While researchers are concerned with a range of properties of these samples when judging sample trustworthiness, this section focuses on one property, namely whether the sample is biologically contaminated.

**Figure 2:**
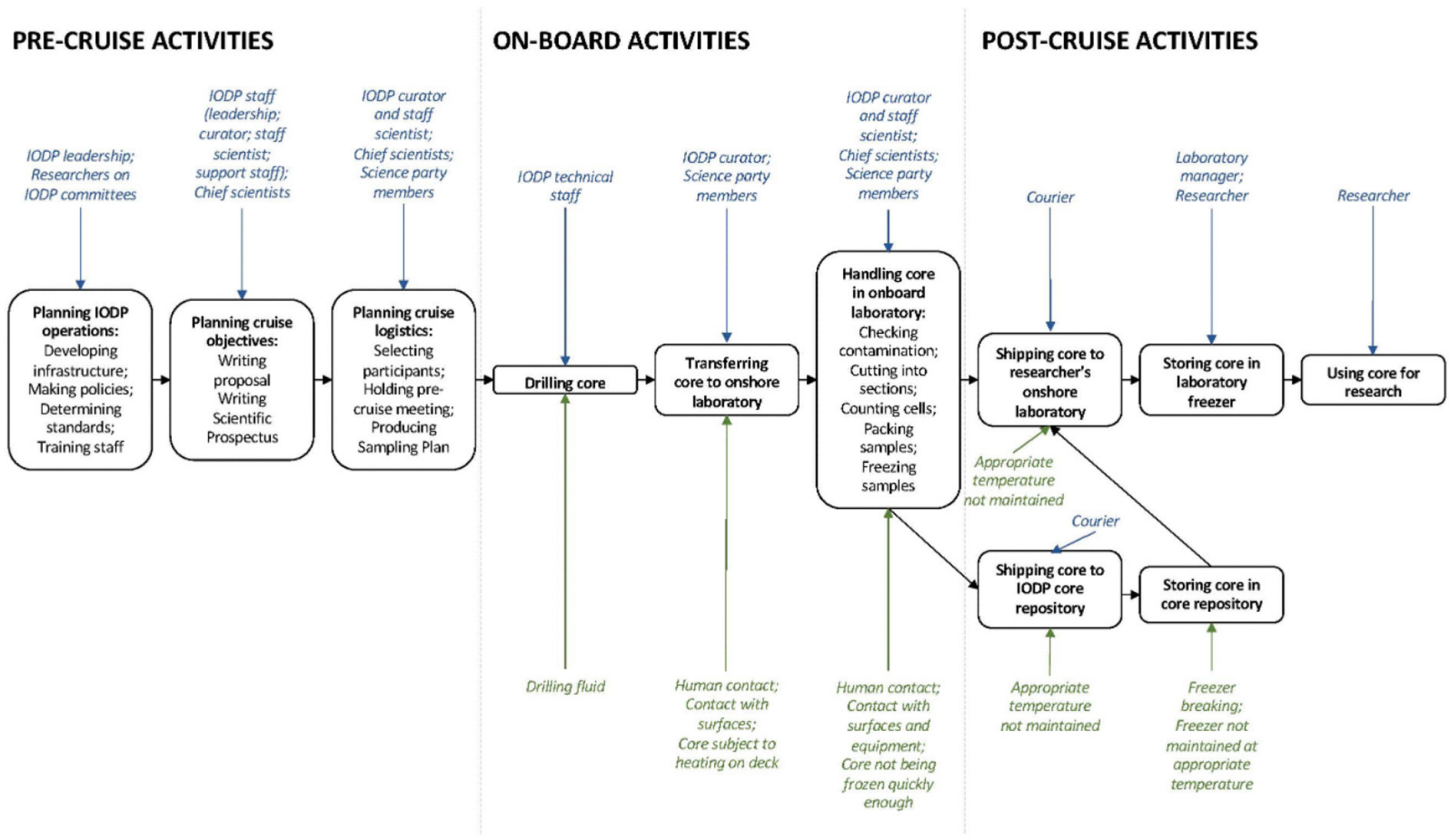
Workflow for production of physical samples on IODP cruises (the blue text above the boxes indicates who is involved at each stage; the green text below the boxes indicates contamination risks that can arise at each stage).

First, however, to show how samples may become contaminated, this section presents an overview of how samples are collected and handled (what stages are involved in this process, who is involved at each stage, and what contamination risks arise at each stage are summarized in Figure 2).

### Production of samples on IODP cruises

IODP operates two large cruise ships, typically hosting five cruises annually, usually nine weeks long. Each cruise visits one site of the ocean and has a specific science mission, such as microbiology or hydrology. This mission will guide the selection of cruise participants (around twenty per cruise, known as the Science Party). Each cruise has one or more chief scientists (known as the scientific leadership team).

Before the cruise, the scientific leadership team sets out the cruise's objectives. Pre-cruise meetings involving the Science Party and IODP representatives determine the Drilling Plan, detailing the quantity and types of cores to be collected and the resulting sample allocation among the Science Party. Participants request samples from specific cores, and their requests are incorporated into the Drilling Plan. Sometimes multiple participants want samples from the same cores, and they must negotiate.

On the cruise, professional IODP staff (technicians and a curator) oversee and perform collection, processing, and preliminary analyses of cores. IODP cruises collect two types of cores: those designated for microbiological analyses and those designated for physical science analyses. This distinction exists for two reasons. One is that cores for microbiological analyses are frozen at −80°C to preserve the microbial communities close to their seafloor state. The second is that cores are subject to possible biological contamination, for instance, from the equipment used to drill and cut cores.

On the ship, separate laboratories handle each type of core. Science party members work twelve-hour shifts to process cores. In the microbiological laboratory, cores are divided into 10 cm long pieces and allocated according to the Drilling Plan. Upon cruise completion, cruise participants send samples back to their onshore laboratories *via* courier. The remaining cores are taken to an IODP repository for long-term storage. Besides the Science Party members, other researchers may also access IODP samples in other ways. For instance, cruise participants may give samples to their students. Researchers may also request samples from the IODP repository.

Researchers in their laboratories conduct multiple analyses on samples (Darch et al., 2015). They extract DNA from cells to characterize the microbial community in a sample (i.e., to determine the quantity and types of microbes in the sample). This DNA is processed to produce DNA sequence data, then compared with sequences in online databases to identify microbial community members. Researchers often compare the microbial community at one site with the communities at other sites. They may also correlate microbiological data with data about the physical composition of samples to understand how the physical environment and microbial communities shape each other.

### How does contamination compromise sample integrity?

Researchers are concerned that DNA sequence data derived from samples only contain data about microbes that live where the sample originated and that these data capture all such microbes. Every researcher interviewed expressed concern about sample contamination by introducing other cells to the sample or changing microbial communities after the sample had been removed from the seafloor.

There are many stages where contamination can occur. Equipment used to drill and bring cores up from the seafloor will almost certainly introduce contamination to the exterior of cores. For instance, the drilling equipment uses seawater as a coolant, which can introduce microbes in the sea to the cores. As cores are brought onto the ship and processed in the laboratory, they are also at risk of contamination from the machines used to process the cores, contact with other cores, contact with humans handling the cores, and contact with surfaces. Resultant samples are also at risk of contamination if they are not frozen quickly enough or not subsequently maintained at an appropriate temperature, as this means microbial communities in the samples are liable to change.

### How do researchers trust that samples are uncontaminated?

Every sample used by a researcher for microbiological analysis has passed through multiple physical environments, has been in contact with many pieces of equipment and physical objects, has been subject to multiple processes, and has been handled by many different people. Each sample is thereby subject to many risks of contamination. The researchers cited many components of IODP cruises when making judgments about whether they trusted that a sample was uncontaminated. Researchers' trust judgments vary according to what components they consider, what facets of each component they emphasize, and what information they use to support their judgments. Further variation occurs in whether and how researchers improvise judgments without desired information.

#### Trust in methods

Many researchers focused on the methods and processes to which a sample had been subjected. Two types of methods were mentioned frequently and are presented here.

*Trust in methods used to check for contamination:* A major contributor to contamination is the penetration of the core by seawater or other materials used as drilling fluid. Contamination checks are tests designed to detect the extent that penetration has occurred. These checks are usually conducted as soon as a core is brought on board the ship. However, checks are sometimes not conducted on cruises. Further, different types of checks exist, with the type used being at the discretion of the chief scientist. Cores that fail contamination checks are not allocated to microbiological work.

Information about contamination checks can help researchers assess a sample's trustworthiness. However, whether a researcher trusts in the result of a check itself is critical in how a researcher assesses the sample's trustworthiness. Multiple factors shape whether a researcher trusts a contamination check. Researchers who participated on the cruise in question were aware, by observation, of whether and what types of contamination checks were conducted. Other researchers sought this information by consulting the post-cruise report. Researchers varied by how they used this information to assess sample trustworthiness. Some researchers focused on whether any contamination check had been conducted, implicitly trusting the check's reliability and accuracy, as illustrated by the following quote:

“When they drilled, they injected a fluorescent dye into the drill fluid, I'm not sure of the exact details, and when the core came into the lab, they did measurements of the core to know how much fluid might have penetrated into the center of the core and the test was negative so we can be sure we have no contamination” (Faculty, #13)

In other cases where the researcher determines that no check had been carried out, they cannot judge whether the core may be contaminated:

“[Another cruise participant] did not want us to use the fluorescent beads that are used as a contamination test in most drilling expeditions because they would be harmful for her samples, I wasn't allowed this time... So, the fluorescent beads, they're the size of cells. So the idea is, is that if you can see these fluorescent beads in your samples, they have been contaminated. So I no longer had a way of tracing contamination” (Postdoctoral Researcher, #6)

However, for other researchers, the mere presence of a check was not enough. Instead, they also focused on the type of check used, trusting some types but not others, as illustrated here:

“Some people had decided in their office that they should use a different protocol for contamination control.. [It] is complete nonsense. It doesn't work... The new detection mechanism they wanted to use was completely bogus” (Faculty, #1)

This quotation is from a researcher with deep expertise in contamination check methods, having published critical reviews of methods and developing their own.

Some researchers encounter one of the following scenarios:

1) They do not know whether any check has been performed on a core;
2) They know that no check has been performed; or
3) They know a check has been performed but do not trust the particular method used.

In these scenarios, researchers do not immediately deem the sample untrustworthy. Instead, they may improvise using other methods. One method is using other available data about the sample, as exemplified in the following quotation:

“One of the things that they were measuring was sulfate. And so, in seawater, sulfate is about 25 millimolar and in my samples it was zero except for in some places. So then when we thought about it and graphed up the results we noticed that the sulfate concentrations were higher at the top of every section. So remember I told you that the sections come up in nine-meter lengths and so at the top of every one it was contaminated with sulfate and that's because the water was getting into the tube as it was coming up. So I was able to eliminate those samples, I'd considered them contaminated” (Postdoctoral Researcher, #6)

In this case, the researcher used certain geochemical datasets as a proxy for checking contamination. The researcher could improvise because she had the necessary scientific expertise (disciplinary training in geochemistry) to understand what these datasets suggested for contamination.

Another method to improvise is for the researcher to perform contamination checks in their onshore laboratory, as explained here:

“I don't know if [the samples are] contaminated or not. And the way for me to identify whether or not they're contaminated now is that I have to have one of my students go through and do the full extraction of the sediments, extract the nucleic acids, isolate those, purify those, get those to send those to be sequenced, and then do sequence analysis on those. Regarding man hours involved, it could be as much as 50 man hours to get them all the way through the process with all the checks and balances that we do, plus an out of the pocket cost of between $200 to $250 per sample to run that and get that through. And I won't know it's contaminated until the tail end, which could be eight, nine, 10 months down the road, whereas if there would have been simple checks done on the ship and simple procedures done on the ship, I would have known, with a better sense of confidence, that these samples were or were not contaminated” (Faculty, #9)

Several interviewees employ this approach. However, as the above quote suggests, it is time- and resource-intensive. Hence, this approach is often not feasible for researchers, such as graduate students, who are resource- and time-constrained.

*Trust in Methods Used to Handle Samples on Ships*: Once a core is brought on deck, many contamination threats arise. One threat is contact with human skin and non-sterilized surfaces or equipment. A second threat is the temperature.

The focus here is on methods for extracting samples from sediment cores. These extractions take place in the onboard laboratory dedicated to microbiological processes. First, the core is divided into shorter segments (typically 10 cm in length); next, samples are taken using a syringe; then, each sample is placed in a bag, and the bag is sealed, labeled, and stored in a freezer. Actions taken to reduce contamination risks include wearing gloves while handling samples, sterilizing syringes and laboratory surfaces, and taking samples from the center of each segment, as the core surface is particularly susceptible to contamination.

The researchers interviewed generally did not express skepticism about the methods themselves. Their concerns instead focused on determining:

- Whether these methods were used in the case of the sample in question; and
- Whether these methods were used properly and without any contingencies arising (e.g., a sample being dropped or not being frozen quickly enough).

Researchers who participated on the cruise from which a sample was collected could use their observations to determine contamination risks [e.g., one interviewee reported they suspected contamination because it was “obvious from the way that samples are treated” (Faculty, #18)].

Researchers who were not on the cruise had a harder time determining the methods used to take samples. Sometimes they check the post-cruise report or online IODP databases [e.g., “that information can be found in the proceedings, so in the proceedings, there is the microbiology part. And usually, [they] state how we cut the samples, by using which kind of instrument or which kind of tools” (Faculty, #10), and, “There is a log that's kept, while on the ship, of what samples were collected, how they were collected, and I consulted that log” (Doctoral Student, #7)].

To find out more, a researcher may ask someone they know who was on the relevant cruise. The interviewee quoted in the previous paragraph supplemented information from the cruise log with testimony from one of the cruise participants:

“So, in talking with [her], because she was on the cruise, she knew what was collected. I had asked her for a list of a few samples that she would recommend to start with, just based upon when they collected them and what they saw” (Doctoral Student, #7)

This interviewee already knew the cruise participant, as they had previously worked in the same laboratory.

#### Trust in people

Many people are involved in collecting and handling samples. A prospective sample user typically only considers, at most, a handful of these individuals when considering sample integrity. These individuals vary according to the prospective user, tending to be those that the prospective user has interacted with directly or those whose reputations are known to the prospective user.

*Trust in a cruise's chief scientist:* An IODP cruise‘s chief scientist plays a leadership role in setting the cruise's main scientific focus and overseeing the Drilling Plan. This Plan determines whether and what contamination checks will be performed, how to respond to unanticipated challenges, and what to include in the post-cruise report.

Some researchers consider the chief scientist when making judgments about sample trustworthiness. These researchers overwhelmingly were those who had already sailed on IODP cruises. They used their observations and experiences with a chief scientist to make inferences about that chief scientist in particular or to generalize to other chief scientists.

*Judgments about the chief scientist focus on competence and personal trustworthiness:* Here, scientific competence refers to awareness of contamination risks and methods to mitigate and measure contamination, while personal trustworthiness relates to whether the prospective sample user believes the chief scientist can be trusted to make decisions in the best interests of deep subseafloor biosphere research over the needs of other domains.

Researchers tend to trust the scientific competence of those chief scientists whose primary disciplinary identity is microbiology and are more skeptical about whether chief scientists from physical science backgrounds are sufficiently aware of the importance of contamination checks. A negative experience with a non-microbiologist chief scientist on a cruise can make a researcher skeptical of non-microbiologist chief scientists in general: “It was a decision made on board by the chief scientist to not use some of our contamination checks… now the samples that I requested, I have a very difficult time to determine whether or not these are contaminated or not. And so that's why we want to take it out of the hands of a chief scientist. We want to educate the chief scientist so that, number one: It's standard, number two: They're better educated as to what happens if you don't do it, and what happens if you do do it, what types of data and what type of better understanding can you have of the subsurface if you support the biology” (Faculty, #9).

This researcher's experience made them skeptical of other chief scientists (e.g., here, the interviewee suggests non-microbiological chief scientists may lack the appropriate education to appreciate contamination issues).

Researchers also made inferences about whether they could rely on the chief scientist to serve the interests of deep subseafloor biosphere research or whether the chief scientist would instead prioritize the interests of another domain. In IODP cruises, domains must share resources and make trade-offs. The following quotation reflects this concern:

“I requested 150 samples... I only got about 50 samples of my 150. And then half of those were from the top and bottom sections of the core, which means most of those are probably not useful to me… [The chief scientists] were trying to do very fine scale chronological data, age-related data to be able to identify specific time points through the geologic record. And so, that's why our samples really got knocked down regarding what we were given” (Faculty, #9)

Here, the researcher links their inability to trust in the samples to the chief scientists' prioritization of geological research ahead of microbiological research.

*Trust in IODP professional staff:* Each IODP expedition sails with a curator and team of technicians. The curator is responsible for organizing and overseeing the process of handling cores and samples on IODP cruises and in the onshore IODP repository. Curators ensure the implementation of IODP procedures for handling and labeling samples, recordkeeping, and conducting standardized tests on a cruise.

Multiple interviewees cited these IODP staff as impacting their judgments of sample trustworthiness in two ways: how their activities affected contamination risks and impacted the cruise records' trustworthiness. These researchers had all participated in IODP cruises previously. Their participation enabled them to get to know at least one curator and a team of technicians and to observe them at work. Based on these observations, these interviewees made inferences about IODP staff in general.

Researchers who cite IODP staff fell into two groups. One group comprised researchers who had served either as chief scientists or who had served on IODP committees, while the other group comprised researchers who had not. Both groups praised the professionalism and dedication of IODP staff [e.g., “There's a curator going out. They are phenomenal. I mean, without them, it would be total chaos” (Faculty, #12)]. Both groups also make judgments about the IODP curators and technicians regarding competence and character but differ regarding how they evaluate the competence and character of IODP staff.

The group without involvement in cruise organization or IODP committees evaluated the competence of the curator and technicians regarding how well they carried out recordkeeping procedures. The following quotation expresses the interviewee's approval of how comprehensively the curator implements labeling and recordkeeping and how that promotes the researcher‘s trust in information about sample provenance:

“They had this curator who takes care of the samples. There is not a single sample that doesn't get a label, they know everything, and I think they do a great job. You can track every sample back, where it was collected, when, who has it, and under what condition it was sampled and preserved” (Faculty, #7)

As well as competence, this group of researchers also used their observations of the IODP staff's demeanor and behavior on cruises to make inferences about staff members' professionalism and dedication, as explained here:

“They had exact formats for the tables, the data entry, it was all there, and they actually had the designated printer for the labels and you have to follow a scheme. It was wonderful. It was so wonderful. And this poor curator, he was so obsessed with it that it made him, I mean, he almost cried when there was a sample that had a wrong label or something…He was always wearing like a hoodie and when things went terribly wrong, he just put up his [hoodie]. I was like, “Oh, no, [he] is upset”” (Faculty, #12)

Those researchers with experience in cruise organization or IODP committees also emphasized another dimension of competence, namely regarding the application of methods to mitigate sample contamination. The researchers interviewed here expressed concern that IODP staff sometimes do not apply adequate methods, as illustrated by the following quotation:

“Get technicians trained by the biologist so that when we are not at sea, the technicians and the trained technician is understanding, the microbial workflow pattern is there. Because the first question you always get is, “Well, how do you know which sampling is really from the subsurface and not from the drill stream, the water column, the ship deck, the drillers themselves?”” (Faculty, #9)

Other interviewees echoed these concerns [e.g., “There‘s a whole bunch of technicians on board but there's no dedicated microbiology technicians…The staff technicians have to be trained. And also, the curators have to know what to do” (Faculty, #11)]. These researchers criticized the lack of appropriate training given to them by IODP. These researchers' involvement with cruise and IODP politics had raised their awareness of how these politics contributed to shortcomings in curatorial training: several interviewees mentioned that they had struggled, in vain, to persuade decision-makers within IODP to devote more resources to training curators.

*Trust in Science Party members:* A third group of people on cruises who impact contamination are the Science Party, comprising around 20 researchers selected to represent a range of scientific backgrounds. However, not every cruise includes a microbiologist. Each Science Party member identifies themselves with a single scientific discipline listed in the post-cruise report. Science party members play multiple roles. One is that they negotiate for cores to be collected at particular sites and to be allocated to them for subsequent research. Another is that they collect data about the core's physical composition and extract samples from cores.

Some interviewees spoke about how Science Party members shape their judgments of samples' trustworthiness. Most Science Party members are unknown to prospective sample users: in these cases, a prospective sample user focused on the disciplinary identity of party members. The interviewees who made judgments based on disciplinary identity were those with a microbiological background who had already participated in IODP cruises. They used these experiences to make inferences about Science Party members regarding competence and character.

Regarding competence, these interviewees found the presence of a microbiologist on a cruise reassuring. They regarded microbiologists as competent to carry out steps necessary to mitigate and check for contamination. Conversely, they were skeptical that a Science Party comprised only of physical scientists would follow these steps. For instance, one interviewee reported that she witnessed physical scientists handling cores with bare hands, which meant she no longer trusted samples from that cruise to be uncontaminated (Faculty, #8, interview unrecorded).

Regarding character, our research subjects evaluated whether they believed Science Party members had the interests of subseafloor biosphere research at heart or whether they instead prioritized the interests of other domains. Only microbiologists could be trusted to advocate for the interests of subseafloor biosphere research to ensure that good quality samples were allocated to microbiology and that contamination checks were carried out, as expressed by the following interviewee:

“The most valuable ones for the microbiologists are the middle sections [of a core]… The problem is, is those middle sections are also those that are best preserved, best maintained and best sampled sections for all the other scientists on board. So we are pulling sections right from the middle, point core material, right from the middle of the core itself, which can disrupt the other field's work. So if there's a microbiologist on board, then the microbiologist has a better chance of lobbying for the right samples.…And there were no contamination checks that were done on any of the cores, so the integrity of each one of my samples is then questioned. If somebody was out there, there'd have been a better chance to lobby for the proper methods” (Faculty, #9)

In other cases, interviewees knew individual members of the Science Party. The most common situation where this happened was students and postdoctoral students working with samples collected from cruises that involved their advisors. In these cases, researchers would trust the recommendation of their advisor:

“So, in talking with [name], because she was on the cruise, she knew what was collected. I had asked her for a list of a few samples that she would recommend to start with, just based upon when they collected them and what they saw” (Doctoral Student, #7)

And:

“Well, obviously contamination's always been an issue.. I actually don‘t know. I wasn‘t on the cruise at all. I don't think [my advisor] was either. She's gone on other cruises. I think she knows generally how it's working. She explained it to me. But I don‘t know about the drilling and a lot of the machines they use now” (Undergraduate Student, #6)

Here, the researchers' lack of cruise experience means they struggle to conceptualize the context in which cores were collected and how contamination may be introduced.

#### How do these factors come together?

Researchers' strategies for trusting in the non-contamination of samples involve many variables. How these variables interact to produce a trust judgment varies between researchers. The range of these interactions is too complex to explain exhaustively here. Instead, this section will focus on how these variables intersect when prospective sample users consider one stage of the lifecycle: whether contamination has occurred from the penetration of drilling fluids into the core. Almost the full range of variables comes into play here.

**Figure 3:**
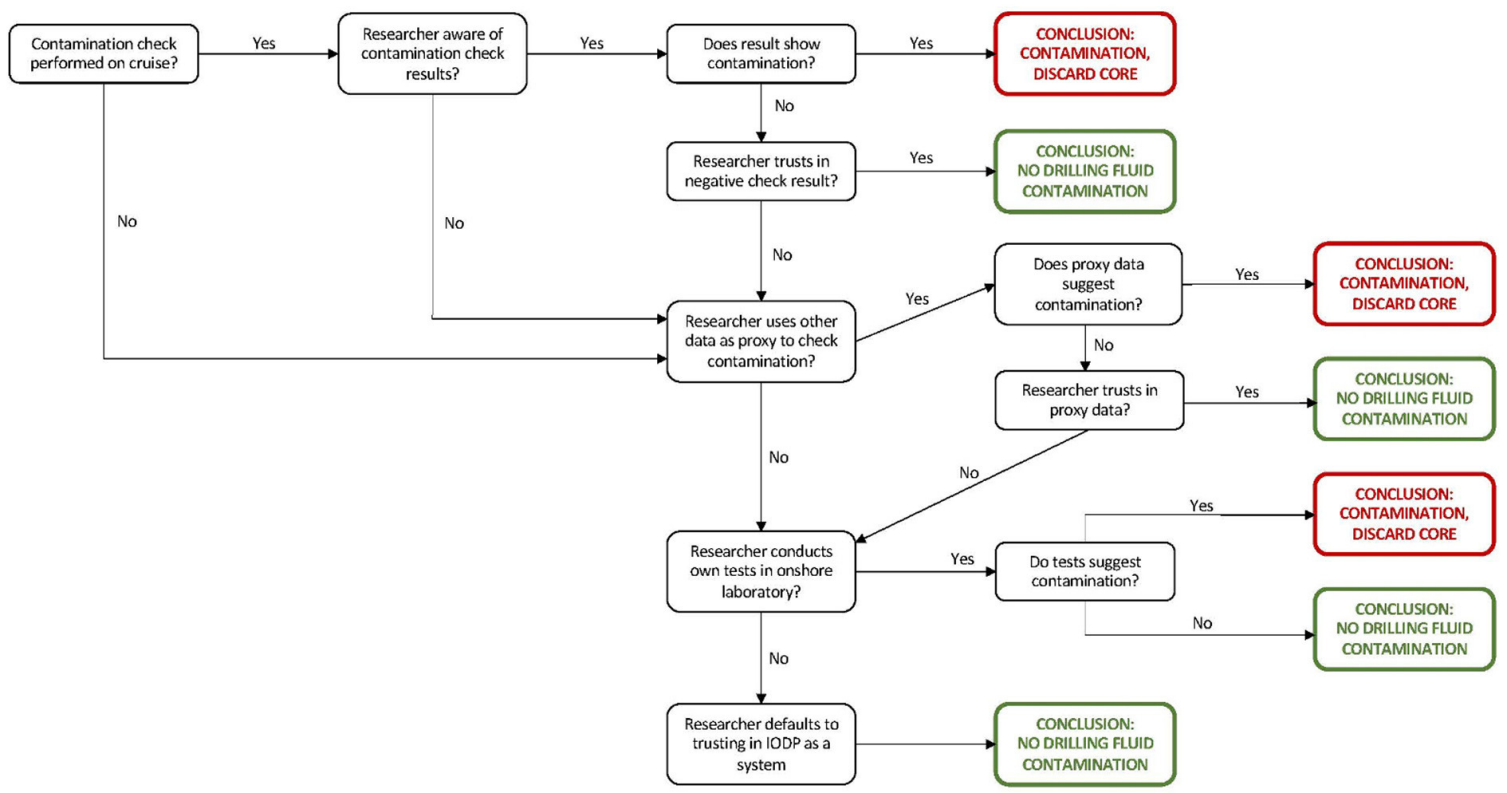
Decision-making process for researcher judging whether an IODP sample cam be trusted to be uncontaminated by drilling fluid.

In general, a researcher's determination of whether they believe a sample has been contaminated by the drilling fluid follows the decision-making process in Figure 3, with the caveats that not all stages are always followed by researchers. For instance, very early-stage researchers (e.g., undergraduate and doctoral students) often work with the samples given to them by their advisors without questioning these samples' trustworthiness.

The variables outlined above influence the researchers‘decision-making process by influencing whether, at each decision node in Figure 3, the determination is “Yes” or “No.” Some of these nodes relate to decisions made by the researcher. Table 2 summarizes how factors that shape researchers' trust strategies influence decision-making at key nodes in Figure 3. The most important information researchers use is whether contamination checks were performed on a sample and the results of these checks. Researchers are not always aware of when a check has been conducted or of the outcome of this check. Early-career researchers who have never been on a cruise are sometimes unaware of the need to conduct checks. Those researchers on the cruise almost certainly know whether a check has been conducted and its outcome. Those researchers who did not participate in the cruise will consult the cruise report, where they are reliant on whether the chief scientist reports the result of a check. If the researcher is aware of the check being performed and its results, they will reflect on the result of that check. If the check suggests the sample is contaminated by drilling fluid, the researcher will discard the sample.

**Table 2:** How factors that shape trust strategies influence decision-making at key nodes in Figure 3.

	**Prior experience with IODP cruise participation and organization**		**Disciplinary background prior to subseafloor biosphere research**	
	**High**	**Low**	**Life sciences**	**Physical sciences**
Researcher aware of contamination check results?	Higher chance of “Yes”	Higher chance of “No”	*N*/A	*N*/A
Researcher trusts in negative check result?	Higher chance of “No”	Higher chance of “Yes”	Higher chance of “No”	Higher chance of “Yes”
Researcher uses other data as proxy to check contamination?	*N*/A	*N*/A	Higher chance of “No”	Higher chance of “Yes”
Researcher conducts own tests in onshore laboratory?	Higher chance of “Yes”	Higher chance of “No”	Higher chance of “No”	Higher chance of “Yes”

A check result that suggests no contamination is not always sufficient for a researcher to conclude that the sample is uncontaminated by drilling fluid. Instead, the researcher must judge whether they trust the negative result means an uncontaminated sample. The decision “Researcher trusts in negative check result?” in Figure 3 breaks down into the following:

1) Does the researcher believe the method of contamination checking used is valid, reliable, and accurate? For most researchers, the answer to this is yes, as they do not question the method's validity. However, those researchers with a particularly deep microbiological expertise may mistrust the method.
2) Does the researcher trust the accuracy of the IODP record reporting the check result? Researchers who have been on cruises are more likely to answer “Yes,” as they often display a high regard for the dedication and professionalism of IODP curators.
3) Does the researcher believe the check was carried out competently? This consideration is more likely to apply to researchers with cruise experience. If the researcher is aware that the chief scientist or a Science Party member is a microbiologist, then they are more likely to conclude “Yes.” If the researcher has not sailed on a cruise, they are unlikely even to ask this question. Researchers who have participated in IODP policymaking or served as chief scientists on a previous cruise are more likely than other researchers to conclude “No” as they are keenly aware of the limitations of IODP training of cruise staff.
4) Does the researcher believe adequate efforts were made on the ship to ensure uncontaminated samples were allocated to microbiological work? This question is only asked by those researchers with prior experience in cruise organization or IODP policymaking, as they know how IODP inter-domain politics compromises the allocation of quality samples to microbiology.

If a researcher does not trust a negative check result or does not have access to contamination check results for a sample, they may improvise. In some cases, they may use other (physical science) data collected on the cruise as a proxy to check for contamination. They are more likely to use these data if they have the physical science background necessary to understand and interpret them. If these proxy data suggest contamination, researchers conclude the contamination risks are too high for them to use the sample. If the proxy data does not suggest contamination, the researcher may (or may not) question whether they trust the proxy physical science data. If researchers are not satisfied that a sample is not contaminated by drilling fluid, they may conduct tests in their onshore laboratory. However, only senior researchers tended to have the resources to do this.

Finally, suppose a researcher has not been able to evaluate sample contamination (typically early career researchers who have not been on cruises). In that case, they are likely to default to trusting in IODP as a system, influenced by the organization‘s reputation and external presentation as conducting well-organized expeditions staffed by highly-trained professionals who carry out standardized workflows according to well-established protocols.

## Discussion

Establishing trust in a sample is a strategic, goal-oriented process. The process does not involve applying a pre-determined set of criteria, invariant across contexts and researchers, to determine whether a sample reaches a particular objective threshold of “trustworthiness.” Instead, researchers are pragmatic. Samples are collected by complex systems comprising multiple human, physical, and non-tangible components. These components, and the interactions between them, can enhance or compromise a sample's integrity. The prospective user operates in uncertainty about most of these components. Nevertheless, researchers still frequently choose to use physical samples, evaluating a sample‘s trustworthiness based on information about a very limited range of these components.

The trust judgments applied by deep subseafloor biosphere researchers to assess the trustworthiness of physical samples resemble, in many ways, the strategies followed by researchers to assess dataset trustworthiness, with some critical differences. Analogous to Yoon's (2017) findings about trust in datasets, this study reveals large variations between researchers regarding their strategies for assessing sample trustworthiness. Researchers also vary regarding the information sources they employ to support their judgments, including their observations and experiences with various IODP processes, domain knowledge, cruise reports and online databases, people they know, and reputational networks.

Researchers' strategies also vary regarding what components of IODP systems they emphasize when considering sample trustworthiness and what facets of each component they emphasize. The components that researchers focus on when making trust judgments about physical samples are similar to those found in studies of how researchers trust in data: the methods used to produce data (Faniel and Jacobsen, 2010; Faniel and Yakel, 2011; Faniel et al., 2012; Fear and Donaldson, 2012), the competence of data producers (Van House, 2002a,b; Jirotka et al., 2005; Faniel et al., 2013), and the systems used to manage and curate datasets (Yakel et al., 2013; Yoon, 2013).

However, the study presented here moves beyond studies of trust in data in several ways. These studies do not account for why particular patterns and trust practices exist in the scientific domain(s) they study and why differences exist between researchers. In the study presented here, researchers' trust strategies vary according to multiple factors: whether they have sailed on a cruise, whether they have served as a (co-) chief scientist of a cruise or participated in IODP policymaking and advisory committees, their disciplinary training, and their professional networks. To account for how these factors shaped variations in trust strategies, it is useful to draw on Latour's concept of the black box (Latour, 1987).

### IODP system as a black box

Whether a researcher had ever sailed on an IODP cruise shapes their trust strategies. An IODP cruise itself can be understood as a black box. Those researchers who had never been on a cruise tended to have rudimentary trust strategies. Although often aware of the risks of sample contamination and that an IODP cruise involves many people and processes, they tend to trust samples from cruises. To these researchers, IODP appears from the outside as a well-organized system, with trained professional staff and well-established standardized policies and procedures that promote the production of high-quality and trustworthy knowledge products.

In contrast, those researchers who had been on IODP cruises had seen inside this black box. They drew on their observations and experiences of people, work, and conditions on IODP cruises to make inferences about the contexts in which samples were produced. Their experiences made them aware of what could go wrong, that procedures were often imperfectly applied, that people were working under pressure and for long hours, and that other contingencies could arise. Compared with researchers who had never been on IODP cruises, these researchers made more detailed judgments about sample integrity, focusing on the people and methods involved.

### Black boxes within the IODP system

Even among researchers who had participated in IODP cruises, variation exists regarding trust strategies. When a researcher opens up the black box of an IODP cruise and has firsthand experience and observation of its inner workings, they encounter more black boxes. In the case here, these other notable black boxes are the methods used to mitigate and check contamination and the organizational activities regarding the cruise and the IODP. Some researchers can see inside some of these black boxes; others cannot—the extent they can see inside them shapes their strategies for trusting in samples.

#### Methods as a black box

Researchers with deep microbiological knowledge are familiar with the contents of the black box of methods for contamination checking. They know that different methods have strengths and weaknesses and that controversy about the “best” method is ongoing. As a result, they consider the contamination checking employed when deciding whether to trust the result of a check. For other researchers, contamination checks remain a black box: from their perspective, it only matters whether a contamination check has been performed.

#### IODP organizational politics as a black box

Opening-up two other black boxes (the organizational activities around the cruise itself and the IODP as an organization) reveals contested political processes around allocating scarce resources between the scientific domains served by IODP. Researchers who have seen inside these black boxes become aware of how these political processes can compromise sample contamination: these insights can shape trust practices. A similar dynamic relates to researchers with experience serving on IODP committees. Those with this experience know the inter-domain politics around scarce IODP resources and how these politics can impact sample contamination risks. As a result, they exercise caution about whether IODP professional staff have been adequately trained.

**Figure 4:**
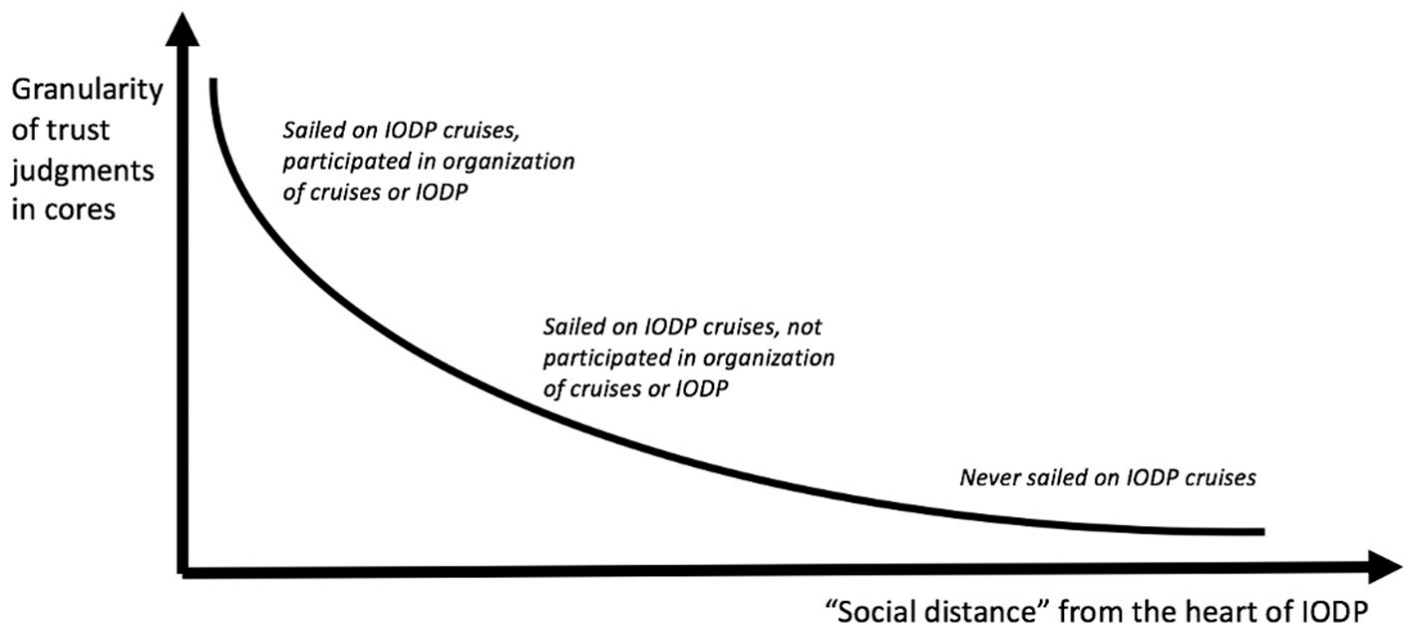
Certainty trough for IODP cruises.

MacKenzie's (1993) certainty trough illustrates how trust judgments vary among researchers. In the case here, “uncertainty” means how wary a researcher is of trusting in a sample. As the study shows, researchers judge sample trustworthiness based on assessing individual components of the system in which the sample was produced and handled. Increasing uncertainty here corresponds to skepticism about an increasing range of components and facets of these individual components. The higher up the y-axis, the more detailed the criteria against which a researcher judges a sample‘s trustworthiness. Figure 4 illustrates how trust judgments vary according to the level of involvement of an individual researcher in IODP. The x-axis metric of “social distance” corresponds to this level of involvement. For this diagram, researchers are crudely divided into three groups, listed from most to least socially distant from the site of sample production:

1) Those who have never sailed in IODP cruises (the most “socially-distant” group). This group is sample users that have never participated in sample production. Given their dependence on IODP cores for their research, they can be understood as “committed to the technological institution/program” (i.e., IODP). As in Mackenzie‘s original diagram, this group generally expressed the lowest level of wariness about sample trustworthiness. Instead, they tended to take for granted that the various policies, procedures, and people on IODP cruises worked smoothly to produce samples fit for use;
2) Those who have sailed on IODP cruises but have not participated in the organization of cruises or IODP itself. These researchers have observed and been involved in the day-to-day work of sample handling and processing onboard cruises. They view many of the people and methods on board a ship as potentially problematic;
3) Those who have participated in the organization of cruises or IODP policymaking. This group is the most “directly involved in knowledge production” because they have observed and participated in organizational inter-domain politics that affect sample production and handling. These researchers display the highest uncertainty of all, problematizing aspects of IODP cruises that other researchers take for granted.

## Conclusions

Findings from this study generalize beyond subseafloor biosphere research. A range of geoscientists shares concerns about physical sample trustworthiness. As with IODP-collected samples, physical samples in the geosciences are often collected on field expeditions whose time-intensive and expensive nature restricts participation. Consequently, researchers must often use samples whose collection and handling they did not observe. Moreover, the infrastructure-intensive nature of many of these expeditions means that decisions and actions taken by many different people can impact sample integrity, and trust judgments may be correspondingly complex.

The focus of work on computational infrastructure to support the circulation of knowledge projects among researchers has largely hitherto been on data and software (Van House et al., 1998; Schuurman and Balka, 2009; Faniel and Jacobsen, 2010; Karasti et al., 2010; Borgman et al., 2012; Howison and Herbsleb, 2013; Mayernik et al., 2013; Rolland and Lee, 2013; Parmiggiani et al., 2015; Young and Lutters, 2017; Mosconi et al., 2019). Physical samples are also critical for research in many earth science disciplines, but significantly less attention has been paid to developing tools and infrastructures to support researchers' use of samples. Existing initiatives have largely focused on improving the findability and accessibility of samples rather than on the provision of information to support judgments of trust in sample integrity and fitness-for-use (Devaraju et al., 2017; Karadkar et al., 2017; Klump et al., 2018, p. 2018; Lehnert et al., 2019).

If the issue of whether a sample is fit-for-use and a researcher's judgment of its fitness-for-use are conceptualized in binary terms, then a researcher will either correctly or incorrectly judge whether a sample is fit-for-use, as illustrated in Table 3. The incorrect judgments that researchers can make when evaluating sample trustworthiness, labeled here for convenience as Error #1 and Error #2, are:

- Error #1: accepting a sample as fit-for-use when it is not fit-for-use (in the subseafloor biosphere case, this means believing a contaminated sample to be uncontaminated);
- Error #2: rejecting a sample as not fit-for-use when it is fit-for-use (in the subseafloor biosphere case, this means believing an uncontaminated sample to be contaminated).

**Table 3:** Possible scenarios when a researcher judges a sample's fitness-for-use.

		**Actual status of sample**	
		**Fit-for-use**	**Not fit-for-use**
Researcher's judgment of sample	Fit-for-use	Correct judgment	Incorrect judgment (Error #1)
	Not fit-for-use	Incorrect judgment (Error #2)	Correct judgment

Each type of error can lead to potentially serious consequences. Error #1 can lead to a researcher using a poor-quality sample and producing (and publishing) incorrect findings, possibly compromising their reputation and misleading other researchers. Error #2 can lead to the researcher throwing away precious and rare samples, losing the opportunity to publish new science and stalling the advancement of their research domain.

Understanding what groups of researchers are particularly susceptible to making each type of error and why is important for formulating and targeting interventions to better support researchers in making judgments about a sample's fitness for use. The findings presented in the Results section are useful in identifying which subseafloor biosphere researchers may be more vulnerable to making each error.

### Reducing researchers' susceptibility to Error #1 based on career status

A group of subseafloor biosphere researchers especially susceptible to making error #1 is early-career researchers, particularly doctoral and postdoctoral researchers, for interconnected factors. These factors stem from limited opportunities to participate in IODP cruises. One consequence is that they cannot advocate for themselves in discussions about sample allocations, meaning they may be more liable to be allocated samples of dubious quality in the first place (Trust in people section). By not participating in cruises, early-career researchers are also more likely to struggle to access information relevant to making judgments about sample trustworthiness, for instance, through not being able to observe contamination checks taking place (Trust in methods section) and not being familiar with individuals involved in curating cores and associated information (Trust in people section). As noted in the Trust in methods section, some researchers who do not participate on cruises can gain relevant information about how cores were handled onboard a cruise by consulting with those who did participate on a cruise, but early-career researchers are less likely to possess the wide professional networks that may enable them to do so readily. Finally, early-career researchers may also be hampered in accessing information to assess sample integrity by being less likely to have control over the material resources (money, a laboratory) necessary to conduct their own contamination tests to supplement those conducted on cruises, as indicated in Trust in methods section.

As well as early-career researchers, other groups of researchers may be liable to making Error 1 for similar reasons. One group is researchers with family commitments that deter them from participating in lengthy or physically demanding expeditions. For instance, a researcher stated in an interview (Faculty #7) they did not sail on cruises because they were reluctant to spend several weeks away from their young child. A second group is researchers from developing countries, who may struggle to access funding to support cruise participation.

Interventions to support these researchers to make better trust judgments promise gains. Early-career researchers may be subject to severe career consequences if they erroneously trust and use a sample whose integrity subsequently becomes compromised. Meanwhile, providing more support to researchers deterred from taking part in expeditions due to family commitments may help address gender gaps in the earth sciences, given that these burdens still often fall primarily on women (Kane, 2018). Finally, better-supporting researchers in developing countries to use physical samples may contribute to realizing one of the stated goals of the Open Science movement to make resources more available to researchers in the Global South (Serwadda et al., 2018).

Proposed measures to help these researchers assess the trustworthiness of datasets focus on what metadata and provenance information needs to be provided alongside datasets. As Trust in methods section demonstrates, many subseafloor biosphere researchers are keenly interested in the methods and tools used to curate cores and perform contamination checks when evaluating sample integrity. Faniel and Yakel (2011) argue that contextual metadata (metadata about methods and tools used to produce and handle datasets and about the environments in which these datasets were collected) can support potential users of datasets to judge dataset integrity. Similarly, collecting and providing rich contextual metadata about the collection and handling of physical samples on cruises should support the decision-making of prospective users of samples.

The provision of such metadata involves addressing critical challenges. One is deciding what information needs to be collected and how to standardize this information (Edwards et al., 2011; Mayernik, 2016). Second is supporting the collection of the necessary information in a field expedition's demanding, time-pressured, and dynamic environment (Lindseth and Baker, 2012). A third is how to support researchers in finding and interpreting these metadata (Willis et al., 2012; Bird et al., 2016). These challenges have been well-addressed elsewhere regarding collecting metadata about datasets.

However, contextual metadata is only a partial solution to the problem of how to support researchers who have never been on expeditions. The purpose of contextual metadata is to help a prospective user of a knowledge product to cognitively reconstruct and visualize the processes that produced that knowledge product. Researchers usually have experience producing their datasets, and they can leverage this experience with contextual metadata to visualize how a dataset was produced. However, a researcher who has never been on a large-scale field expedition may struggle to visualize a process they have never observed, even with rich contextual metadata. How can they conceptualize how decisions were made and the effect of these decisions in a context they have never experienced?

For a researcher who has never taken part in a large-scale field expedition, alphanumeric contextual metadata may not suffice. Instead, large-scale infrastructures might consider including video clips of various processes carried out on a particular sample alongside text-based contextual metadata. Some videos may be annotated to explain what is being seen. Further, these infrastructures should also consider including training sections on their websites for researchers that provide narrated/annotated videos of standard processes carried out on field expeditions.

### Reducing researchers' susceptibility to Error #1 based on disciplinary background and identity

A researcher's disciplinary background can also affect how they can make trust judgments in samples. Those without the appropriate disciplinary training may tend to be more trusting in the methods used to produce and handle samples, thereby increasing their susceptibility to Error #1. In contrast, those with appropriate disciplinary backgrounds may be more aware of shortcomings and controversies relating to the chosen methods, making more detailed judgments of the trustworthiness of the samples (as illustrated by the contrasting quotes given by Faculty #14 and Faculty #1 in the Trust in methods section).

Disciplinary identity can also play a role in determining a prospective sample user‘s awareness of inter-domain politics and their impact on sample integrity. Some researchers cycle between scientific domains, whereas others identify closely with a single domain. The latter group is more likely to be involved in the negotiations and politics around resources allocated to their domain. Earth science's focus is increasingly on studies of global systems, which require collecting and integrating knowledge produced from a range of specialisms. Supporting researchers to use knowledge products produced and handled using methods that originated in other domains is a tough challenge. At the moment, metadata schemas for physical samples only allow for brief descriptions of the methods used, conveying to a prospective sample user only that a particular method has been used rather than whether the method is widely-accepted or whether debates persist around its reliability, validity, accuracy, or unbiasedness (Golodoniuc et al., 2016). A domain outsider not versed in these debates may end up placing more confidence than is warranted in the method. To address this situation, contextual metadata should convey whether the method is widely accepted or whether doubts exist about its suitability.

### Reducing researchers' susceptibility to Error #2

In most earth science disciplines, expeditions to collect physical samples can be expensive and resource-intensive. Rejecting and discarding perfectly good samples due to Error #2 can prove costly. In the case here, experiences of subseafloor biosphere researchers in IODP internal politics and cruise organization tend to increase their skepticism of sample integrity, as noted by Faculty #9 in Trust in methods section. In some cases, this skepticism makes researchers warier about using samples. In other cases, it leads researchers to conduct lengthy and expensive tests in their laboratories. This increased skepticism may sometimes be misplaced, leading to the wasting of scarce resources.

Mitigating the risk of Error #2 among these researchers requires enhancing their confidence that decisions about how to build and operate infrastructures incorporate the interests of their domain. Decisions affecting all components of a particular infrastructure should involve substantive participation from all relevant domains served by that infrastructure. One component is building tools and instruments to collect data and metadata about samples and developing systems for accessing and interpreting these forms of information. Devising and implementing standards is also a critical part of building infrastructure (Edwards et al., 2013) and is often a site of contestation between domains and communities because competing options for standards often serve the interests of different domains in varying ways (Darch, 2016). The resolution of standards-making can result in some communities being disenchanted. Participatory approaches to standards-making may be useful (Yarmey and Baker, 2013).

Representatives of all domains served by an infrastructure might also be involved in devising and even training the human infrastructure of cyberinfrastructure (Jirotka et al., 2013). For instance, some moves were made by subseafloor biosphere researchers during 2013-15 to develop training for IODP curators and technicians to carry out routine contamination checks and cell-counting procedures on all IODP cruises (although this training was not eventually implemented). Infrastructural projects might also pay attention to the representation of the domains they serve in field expeditions. For instance, IODP not including a microbiologist as standard on every cruise seems to undermine trust in physical samples because other researchers worry about the absence of a microbiologist to advocate for subseafloor biosphere research's interests in core allocations (Trust in people section), potentially increasing the risk of Error #2.

In many scientific domains, research datasets are generated from physical samples collected from large infrastructural projects comprising multiple physical, technical, and human components. Supporting researchers in evaluating the trustworthiness of these samples promises critical gains, including promoting the circulation and reuse of datasets generated from samples, enhancing the scientific integrity of research findings based on samples, and enabling researchers to use samples they did not collect confidently. The case study in this paper reveals that supporting researchers' trust judgments is not easy. Even within a single, small research domain, researchers' strategies for trusting in samples vary considerably, with different researchers focusing on different components of the large infrastructural project where samples were collected. Challenges multiply as samples are transferred to researchers with different levels of insight into the inner workings of this infrastructure and to researchers with divergent scientific expertise.

Supporting researchers to evaluate the trustworthiness of samples requires the development of tools and approaches that enable researchers to conceptualize the processes and contexts in which samples are produced and handled. Metadata schemas need to be expanded to capture more and different forms of information (e.g., video). Meanwhile, inclusive, participatory approaches to tool development will enable more researchers to get to the core of the matter of determining whether a physical sample is trustworthy.

## Data availability statement

The datasets presented in this article are not readily available because the data in this article is human subjects data, and cannot be released given their sensitive nature and the infeasibility of de-identification. Requests to access the datasets should be directed to ptdarch@illinois.edu.

## Ethics statement

The studies involving human participants were reviewed and approved by UCLA North General Institutional Review Board (IRB#10-000909-CR-00002). The patients/participants provided their written informed consent to participate in this study. Written informed consent was obtained from the individual(s) for the publication of any potentially identifiable images or data included in this article.

## Author contributions

The author confirms being the sole contributor of this work and has approved it for publication.

## Funding

This research was funded by the Alfred P. Sloan Foundation (Awards: #20113194 and #201514001).

## Conflict of interest

The author declares that the research was conducted in the absence of any commercial or financial relationships that could be construed as a potential conflict of interest.

## Publisher's note

All claims expressed in this article are solely those of the authors and do not necessarily represent those of their affiliated organizations, or those of the publisher, the editors and the reviewers. Any product that may be evaluated in this article, or claim that may be made by its manufacturer, is not guaranteed or endorsed by the publisher.
